# *Notes from the Field:* Mumps Outbreak — Alaska, May 2017–July 2018

**DOI:** 10.15585/mmwr.mm6733a6

**Published:** 2018-08-24

**Authors:** Amanda Tiffany, Drew Shannon, Willy Mamtchueng, Louisa Castrodale, Joseph McLaughlin

**Affiliations:** ^1^Epidemic Intelligence Service, CDC; ^2^Alaska Division of Public Health, Section of Epidemiology, Anchorage, Alaska; ^3^Department of Health and Social Services, Municipality of Anchorage, Anchorage, Alaska.

In May 2017, the Alaska Section of Epidemiology (SOE) was notified of an Anchorage resident with laboratory-confirmed mumps who reported exposure to an out-of-state visitor with mumps-like symptoms. Another seven laboratory-confirmed cases were reported in late July and August; all were in Anchorage residents, mostly in persons who self-identified as Native Hawaiian or other Pacific Islander (NH/PI). In response, SOE disseminated educational materials and recommended that all Alaskans ensure that they were up to date on their measles-mumps-rubella (MMR) vaccinations. Cases were classified as suspected, probable, or confirmed according to the Council of State and Territorial Epidemiologists case definition (*1*).

On November 15, with 56 confirmed and probable mumps cases identified (including 82% among NH/PI, who represent 4.8% of the Anchorage population), SOE recommended a third dose of MMR vaccine (MMR3) for persons at increased risk for acquiring mumps, such as persons participating in group settings (e.g., school, daycare, church) where mumps cases were identified or any persons who self-identified as NH/PI, if at least 5 years had passed since their second MMR dose (*2*). Despite this recommendation, cases continued to occur among persons at increased risk and among persons without documented epidemiologic links to other cases. Consequently, on December 28, 2017, when 138 cases had been reported, the MMR3 recommendation was expanded to all Anchorage residents. On February 22, 2018, with 247 cases reported, and cases continuing and occurring statewide among persons with indeterminate epidemiologic links and no history of in-state or out-of-state travel, the recommendation was extended to all 737,080 Alaska residents.

Concurrently, SOE and the Anchorage Department of Health and Human Services coordinated community outreach in collaboration with local partners to offer targeted vaccination clinics, presentations, and media campaigns to raise awareness about the outbreak and the importance of vaccination. Since November 15, 2017, when the first MMR3 recommendation was made, through July 31, 2018, the average number of MMR doses administered in Anchorage (where most of the outreach was focused) increased by 136% to 461 per month, from 195 per month before the recommendation (November 1, 2016–November 15, 2017) (p = 0.001) (Figure).

**FIGURE:**
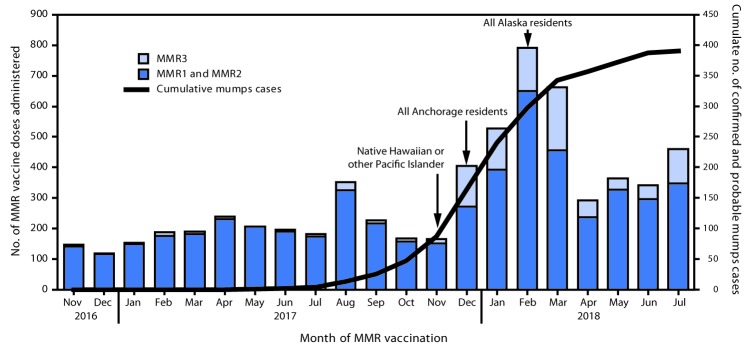
Cumulative number of confirmed and probable mumps cases and MMR vaccine doses administered,* by dose number and month of vaccination — Anchorage, Alaska, November 2016–July 2018 **Abbreviations:** MMR = measles-mumps-rubella vaccine; MMR1 = first MMR dose; MMR2 = second MMR dose; MMR3 = third MMR dose. * Arrows indicate the month during which MMR3 recommendation was made for specific populations.

As of July 31, 2018, the outbreak is ongoing, with 391 confirmed and probable cases reported; 94% of cases have been in Anchorage residents. The median age of patients was 25 years (range = 3 months–79 years) and 193 (49%) self-identified as NH/PI. Overall, 162 (41%) patients had received ≥2 MMR doses before symptom onset, 51 (13%) received 1 dose, and 15 (4%) had not received MMR; vaccination status was unknown for 163 (42%) patients.

Compared with mumps outbreaks in discrete populations such as universities where the population at risk is well defined, community outbreaks pose unique challenges. Following updated Advisory Committee on Immunization Practice recommendations (*3*), a third MMR dose was recommended for persons at increased risk for acquiring mumps as defined by the epidemiologic data. However, as the outbreak evolved, it became more difficult to determine who was at increased risk. Group-specific MMR3 recommendations were challenging for clinicians to implement when faced with uncertainty about whether their patients participated in group settings where mumps was circulating. In response, SOE implemented a stepwise expansion of its MMR3 recommendation that eventually included all Alaskans. Evaluation of Alaska’s response to the mumps outbreak, including the impact of MMR3 recommendations on MMR uptake, is ongoing. Disseminating information through social media, working with community groups, and vaccination clinics have been important in raising awareness and increasing vaccine uptake.
